# nipalsMCIA: flexible multi-block dimensionality reduction in R via nonlinear iterative partial least squares

**DOI:** 10.1093/bioinformatics/btaf015

**Published:** 2025-01-12

**Authors:** Max Mattessich, Joaquin Reyna, Edel Aron, Ferhat Ay, Misha Kilmer, Steven H Kleinstein, Anna Konstorum

**Affiliations:** Department of Engineering Sciences and Applied Mathematics, Northwestern University, Evanston, IL 60208, USA; Center for Autoimmunity and Inflammation, La Jolla Institute for Immunology, La Jolla, CA 92037, USA; Bioinformatics and Systems Biology PhD Program, University of California, San Diego, La Jolla, CA 92093, USA; Program in Computational Biology and Bioinformatics, Yale University, New Haven, CT 06510, USA; Center for Autoimmunity and Inflammation, La Jolla Institute for Immunology, La Jolla, CA 92037, USA; Bioinformatics and Systems Biology PhD Program, University of California, San Diego, La Jolla, CA 92093, USA; Department of Pediatrics, University of California, San Diego, La Jolla, CA 92093, USA; Department of Mathematics, Tufts University, Medford, MA 02155, USA; Program in Computational Biology and Bioinformatics, Yale University, New Haven, CT 06510, USA; Department of Pathology, Yale School of Medicine, New Haven, CT 06510, USA; Department of Immunobiology, Yale School of Medicine, New Haven, CT 06510, USA; Department of Pathology, Yale School of Medicine, New Haven, CT 06510, USA; Center for Computing Sciences, Institute for Defense Analyses, Bowie, MD 20715, USA

## Abstract

**Summary:**

With the increased reliance on multi-omics data for bulk and single-cell analyses, the availability of robust approaches to perform unsupervised learning for clustering, visualization, and feature selection is imperative. We introduce nipalsMCIA, an implementation of multiple co-inertia analysis (MCIA) for joint dimensionality reduction that solves the objective function using an extension to Nonlinear Iterative Partial Least Squares. We applied nipalsMCIA to both bulk and single-cell datasets and observed significant speed-up over other implementations for data with a large sample size and/or feature dimension.

**Availability and implementation:**

nipalsMCIA is available as a Bioconductor package at https://bioconductor.org/packages/release/bioc/html/nipalsMCIA.html, and includes detailed documentation and application vignettes.

## 1 Introduction

Multiple co-inertia analysis (MCIA) is a member of the family of joint dimensionality reduction (jDR) methods that extend unsupervised dimensionality reduction techniques such as Principal Component Analysis (PCA) and Nonnegative Matrix Factorization (NMF) to datasets with multiple data *blocks* (alternatively called *views*) (Tenenhaus and Tenenhaus 2011, Cantini *et al.* 2021). Such methods, also known as multi-block or multi-view analysis algorithms, are becoming increasingly important in the field of bioinformatics, where data is often collected simultaneously using multiple *omics* technologies such as transcriptomics, proteomics, epigenomics, metabolomics, etc. (Karczewski and Snyder 2018). MCIA has the capability to simultaneously derive both global and block-level low-dimensional sample embeddings for use in visualization and clustering, as well as provide valuable information in regards to the block- and feature-level contributions to the embedding, allowing for straightforward downstream analysis of contributing biological pathways.

**Figure 1.**
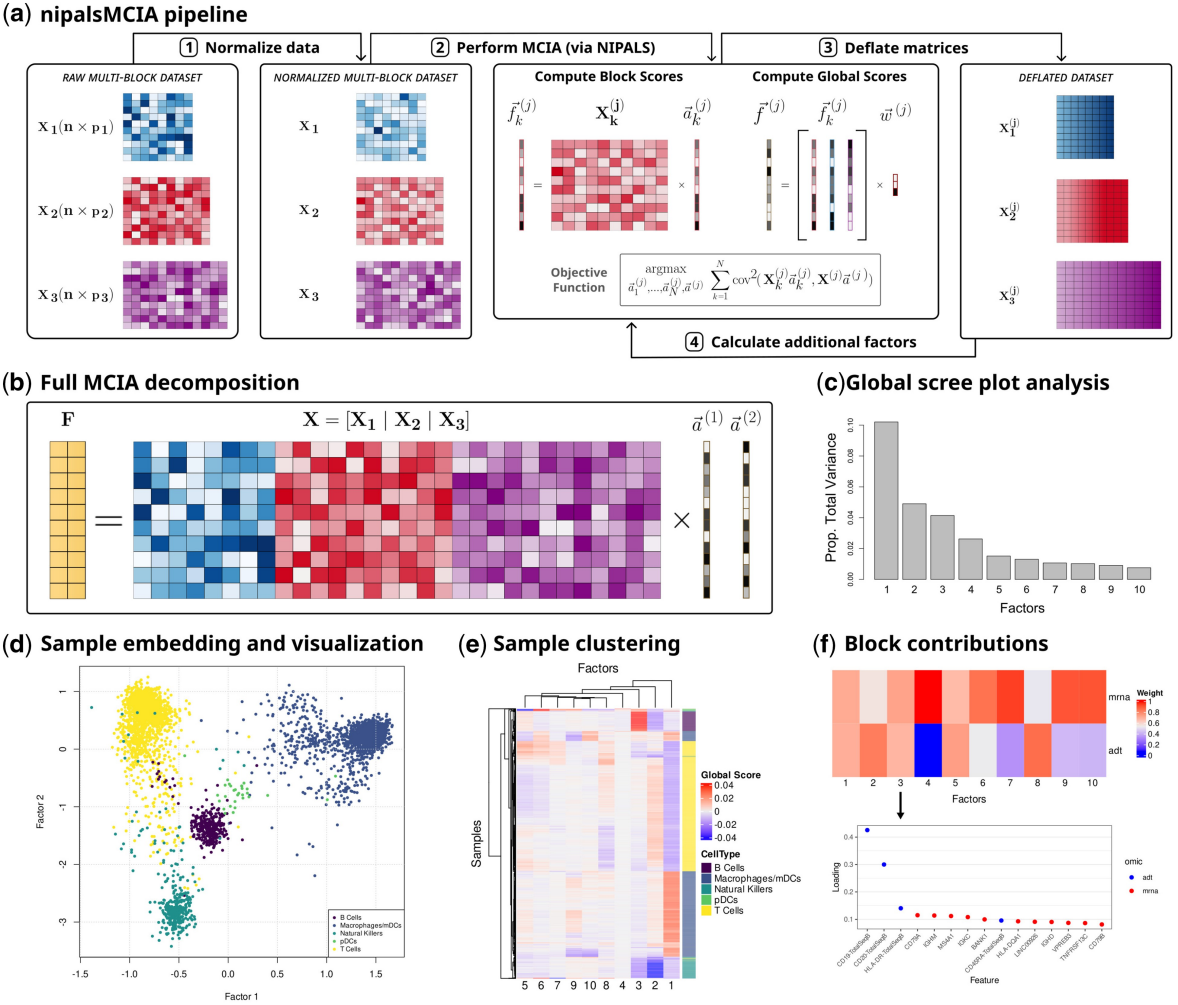
nipalsMCIA workflow overview with sample decomposition of single-cell multi-omic data. (a) A summary of the nipalsMCIA pipeline for performing MCIA. Data blocks are normalized before scores and loadings are computed to satisfy the objective function. Higher-order results are computed after the data has been deflated with the current scores or loadings. (b) Factors (global scores) (F) can be calculated from the global data matrix (X) and global loadings (A). (c) Scree plot for the proportion of variance explained by each factor in PBMC single-cell multi-omics (mRNA and ADT) data (10x Genomics 2019). (d) Low-dimensional global embedding of cells by the first two factors. (e) Clustering of cells using all global factor scores via hierarchical clustering. (f) Block contributions to each factor can be visualized, and a global loadings vector can be plotted to identify the top features associated with a factor.

Here, we present a new implementation in R/Bioconductor of MCIA, nipalsMCIA, that uses an extension with proof of monotonic convergence of Nonlinear Iterative Partial Least Squares (NIPALS) to solve the MCIA optimization problem (Hanafi *et al.* 2011). This implementation shows a significant speed-up over existing Singular Value Decomposition (SVD)-based approaches for MCIA (Meng *et al.* 2014, 2019) on large datasets. Furthermore, nipalsMCIA offers users several options for pre-processing and deflation to customize algorithm performance, methodology to perform out-of-sample global embedding, and analysis and visualization capabilities for efficient results interpretation. We show applications of nipalsMCIA to both bulk and single-cell multi-omics data. The overall workflow, including optimization steps and an application to single-cell multi-omics data, for nipalsMCIA is outlined in Fig. 1.

## 2 MCIA: theoretical background

### 2.1 Notation and preliminaries

Scalars, vectors, and matrices are represented in lowercase script (*a*), lowercase script with a vector symbol ($\vec{a}$), and bold uppercase script (A), respectively. The *i*th column vector of a matrix A is denoted ${\vec{a}}^{\left(i\right)}$. Since we are evaluating several datasets (termed *blocks*) simultaneously, the sample-by-feature data matrix for the *k*th block is labeled as $X_{k}$. We denote the column-wise concatenation of *N* data blocks as the “global” data matrix $X=[X_{1}|\ldots |X_{N}]$. Similarly, we take $X^{\left(j\right)}=[X_{1}^{\left(j\right)}|\ldots |X_{N}^{\left(j\right)}]$ as the data matrix at any deflation step j=1,…,R. We use the convention that $X^{\left(1\right)}$ and $X_{k}^{\left(1\right)}$ correspond to the original (un-deflated) data matrices.

### 2.2 Loadings and scores

MCIA extends the concept from PCA of deriving principal components (which we term *scores*) and loadings (which we also term *loadings*) to the multi-block setting. The loadings are a set of optimal axes in feature space, while the scores are the projection coefficients of the samples onto these axes. Unlike PCA, MCIA generates two types of scores and loadings, one set for all the data (*global* scores/loadings), and the other for the individual omics (*block* scores/loadings). The number of scores/loadings generated is equal to the dimension *R* of the MCIA embedding of the data.

Originally, the optimization criteria for MCIA were presented using the concept of *statistical triplets* (Chessel and Hanafi 1996, Meng *et al.* 2014). The criteria can equivalently be represented as a parameterized member of the Regularized Canonical Correlation Analysis (RGCCA) family of multi-variate dimensionality reduction methods (Tenenhaus and Tenenhaus 2011, Meng *et al.* 2016), which is consistent both with the optimization criteria that is solved by an extension of the NIPALS algorithm (Hanafi *et al.* 2011), and via direct eigendecomposition.

In the multi-block dataset, each block must share the same *n* samples (rows), but the number of features (columns) $p_{k}$ in each block *k* can vary. nipalsMCIA generates distinct block-level scores ($F_{k}^{n\times R}$) and loadings ($A_{k}^{p_{k}\times R}$), and global scores ($F^{n\times R}$) and loadings ($A^{p\times R}$), where $p=\sum_{k=1}^{N}p_{k}$, and *R* is the dimension of the embedding.

The objective function solved by MCIA (with constraints specific to nipalsMCIA) is


(1) $$\begin{array}{c} \underset{{\vec{a}}_{1}^{\left(j\right)},\ldots ,{\vec{a}}_{N}^{\left(j\right)},{\vec{a}}^{\left(j\right)}}{\text{argmax}}\;\;\sum_{k=1}^{N}{\text{cov}}^{2}(X_{k}^{\left(j\right)}{\vec{a}}_{k}^{\left(j\right)},X^{\left(j\right)}{\vec{a}}^{\left(j\right)})\\ {\left({\vec{a}}_{k}^{\left(i\right)}\right)}^{T}{\vec{a}}_{k}^{\left(j\right)}=\delta_{ij},\;{\vec{f}}^{\left(j\right)}=\left[{\vec{f}}_{1}^{\left(j\right)}\ldots {\vec{f}}_{N}^{\left(j\right)}\right]{\vec{w}}^{\left(j\right)}, \end{array}$$

where ${\vec{w}}^{\left(j\right)}={\left(w_{1}^{\left(j\right)},w_{2}^{\left(j\right)},\ldots ,w_{N}^{\left(j\right)}\right)}^{T}$ is a vector of block contributions to the *j*th order global score, with constraint $||{\vec{w}}^{\left(j\right)}|{|}_{2}=1$ for all orders j=1,…,R as in Hanafi *et al.* (2011), and $\delta_{ij}$ is the Kronecker delta function. Equation (1) is solved separately for each order (*j*) up to the dimension of the embedding, *R*. While there exist minor differences in the constraints for Equation (1) depending on the implementation, these differences only impart a different scaling for the results (Supplementary Materials S1). The block scores $\left\{{\vec{f}}_{k}^{\left(1\right)},{\vec{f}}_{k}^{\left(2\right)},\ldots ,{\vec{f}}_{k}^{\left(R\right)}\right\}$ represent an *R*-dimensional embedding of the samples in the orthonormal set of block loadings vectors for block *k*. This contrasts with Consensus PCA (CPCA), which solves for the same objective function as MCIA, but with an orthogonality constraint on the global scores instead of the block loadings (Hassani *et al.* 2013). In nipalsMCIA, users can choose to use either method.

### 2.3 NIPALS strategy for computing MCIA

Currently available methods for computing MCIA [e.g. Omicade (Meng *et al.* 2014) and MOGSA (Meng *et al.* 2019)] perform direct eigendecomposition from the principal components of the covariance matrix (see Tenenhaus and Tenenhaus 2011). Conversely, the implementation in nipalsMCIA uses an extension of NIPALS method (Hanafi *et al.* 2011). NIPALS was first introduced as an iterative (power) method to estimate principal components (Wold 1966, Miyashita *et al.* 1990), and later extended to the multi-block setting (Wold *et al.* 1987). A modification of the multi-block algorithm was proven to have monotonic convergence (Hanafi *et al.* 2011). Since the NIPALS procedure is iterative, it does not require an eigendecomposition. The computational cost of the iterative method used in nipalsMCIA is O(nmS), where *n* is the number of samples, *m* the total number of features, and *S* the number of iterations per order. In practice S≪min(n,m) for larger datasets, thereby giving an advantage to nipalsMCIA over eigendecomposition-based methods, which have complexity O(nmmin(m,n)) or $O(\min{\left(n,m\right)}^{3})$, depending on the implementation. Moreover, the use of nipalsMCIA imparts additional cost savings with respect to memory usage. In nipalsMCIA, the stable multi-block extension to NIPALS (Hanafi *et al.* 2011) is implemented with deflation options for both MCIA and CPCA, and variance explained by each component is also calculated without reference to an eigendecomposition.

## 3 Usage and functionality

Since MCIA is designed to handle multiple omics data blocks, pre-processing options are available both at within- and whole-block levels. The latter is recommended to account for potential disparities in block size.

### 3.1 Analysis and interpretation

The nipals_multiblock function is used to run MCIA in nipalsMCIA. The function outputs an object of the NipalsResult class, which includes the global scores and loadings, block scores and loadings, the global score eigenvalues, and the block score contributions vector for all orders up to the maximum specified via the num_PCs argument. The global scores represent the projection of the multi-block data in the reduced space, and can be plotted with or without corresponding block scores (Fig. 1d). The contribution of each block to the global score can be easily visualized, along with high-scoring features (Fig. 1f).

Vignettes providing full analysis pipelines using nipalsMCIA for bulk and single-cell data are available within the package. The example bulk data is a subset of the National Cancer Institute 60 tumor-cell line screen (NCI60 data) (Shoemaker 2006, Meng *et al.* 2016). It includes RNA-Seq, miRNA, and protein data from 21 cell lines that correspond to three cancer subtypes (brain, leukemia, and melanoma). The single-cell data is sourced from 10x Genomics and includes both gene expression and cell surface antibody (ADT) data of peripheral blood mononuclear cells (PBMCs) from a single healthy donor (10x Genomics 2019). The single-cell analysis vignette includes instructions on how to obtain, process, and prepare the dataset for nipalsMCIA, along with a demonstration of the ability of nipalsMCIA to effectively cluster annotated cell types in a computationally efficient manner.

### 3.2 Out-of-sample embedding

It can be shown that the loadings vectors generated by MCIA on a dataset X represent linear combinations of the original features of X (Supplementary Materials S1). Therefore, after computing MCIA on a training dataset, one can use the associated loadings vectors to predict global embeddings for a test dataset of new observations of the same features. nipalsMCIA provides the predict_gs function to facilitate this task.

This can be valuable for testing the quality of the embedding, as well as embedding new data without rerunning the decomposition. We provide a vignette in the package showing how this can be done using the NCI60 dataset, using 70% of the data to train the model, and then deriving global scores for the remaining 30%.

## 4 Computation time comparison for MCIA algorithms

We used four datasets to compare the performance of nipalsMCIA with two other implementations for MCIA: MOGSA and Omicade. The datasets are composed of the NCI60 data (21 samples measuring mRNA, miRNA, and protein), single-cell peripheral blood mononuclear cells (PBMCs) data (4193 cells measuring mRNA and ADT) (10x Genomics 2019), the same data filtered for the top 2000 most variable genes, and a large single-cell dataset of inflammatory skin disease (>140K cells measuring mRNA and ADT) available through the Human Cell Atlas (HCA) data portal (Liu *et al.* 2022). We note that there exist data modalities, especially in epigenomics (e.g. ATAC, Hi-C, and methylation), where feature dimension is in the >100K range, and feature selection is not typically performed (Luecken *et al.* 2022); moreover downstream investigations such as differential expression and pathway analysis are generally performed upon the entire set of features for a given omic. Therefore, comparing implementation performance without feature filtering provides a useful benchmark for such applications.

Data pre-processing was standardized across all algorithms and a decomposition for 10 factors was performed across all datasets and implementations. All experiments were performed in R 4.3.0 on a MacBook with 3.2 GHz and 16 GB RAM. The dimensions of the datasets and performance are shown in Table 1. We observe that while MOGSA has slightly faster performance than nipalsMCIA and Omicade on the smaller NCI60 dataset, nipalsMCIA is an order of magnitude faster for all single-cell datasets, even when using the “fast svd” option in MOGSA. Additionally, we note that we used a stringent convergence tolerance for nipalsMCIA (tol=1e−12), therefore, a further speed-up can be obtained by setting a lower tolerance.

**Table 1.** Computation time (in seconds) comparison for different MCIA implementations and datasets.

	Dataset (dimension)			
	NCI60	10x single cell (filtered)	**10x single cell (full)** ^a^	**HCA single cell** ^a^
Implementation	21 × (12 895, 547, 7016)	4193 × (2000, 32)	4193 × (33 538, 32)	1 45 810 × (2000, 35)
nipalsMCIA	2.3	15.32	289.46	671.78
MOGSA	0.53	519.55	NA	NA
MOGSA (fast svd)	0.38	434.84	13 840.66	DNC^b^
Omicade	2.66	1089.53	NA	NA

a Due to the slow performance of MOGSA without the fast svd option and Omicade, only nipalsMCIA and MOGSA with the fast svd option were tested for the larger single-cell datasets.

b MOGSA (fast svd) ran into a memory error and did not converge (DNC).

The convergence properties of nipalsMCIA guarantee that embeddings will be equivalent to those computed using the methods that rely on eigendecomposition (up to scaling and orientation). The speedup offered by nipalsMCIA thus opens up capabilities for practical deployment of nipalsMCIA on a larger variety of datasets, including high-dimensional single-cell data.

## 5 Discussion

The accessibility of next-generation sequencing and other high-throughput biological assays are resulting in an increase of multi-block (or multi-modal) datasets (Perez-Riverol *et al.* 2017, Vasaikar *et al.* 2018, Conesa and Beck 2019, Mohammadi-Shemirani *et al.* 2023). Analysis of these data are facilitated by the application of joint dimensionality reduction methods such as MCIA. nipalsMCIA is a comprehensive R package that implements MCIA in a highly efficient manner using the NIPALS algorithm. The package features various pre-processing and analysis options, is much faster for large input datasets compared with existing packages, supports the projection for out-of-sample scores, and offers visualization options for scores and top-magnitude loadings at each order.

## Supplementary Material

btaf015_Supplementary_Data
